# Diverse Health, Gender and Economic Impacts from Domestic Transport of Water and Solid Fuel: A Systematic Review

**DOI:** 10.3390/ijerph181910355

**Published:** 2021-10-01

**Authors:** Erica W. Ho, Sophia Strohmeier-Breuning, Madeleine Rossanese, Dana Charron, David Pennise, Jay P. Graham

**Affiliations:** 1 Berkeley School of Public Health, University of California, 2121 Berkeley Way, Berkeley, CA 94720, USA; erica_ho@berkeley.edu; 2 Department of Public Health Sciences, UC Davis School of Medicine, University of California-Davis, Davis, CA 95616, USA; sstrohmeier@ucdavis.edu; 3 Berkeley Air Monitoring Group, Berkeley, CA 94704, USA; mrossanese@berkeleyair.com (M.R.); dcharron@berkeleyair.com (D.C.); dpennise@berkeleyair.com (D.P.)

**Keywords:** water, solid fuel, gender, accessibility, heavy load carrying, pain

## Abstract

(1) Background: Water and solid fuel collection and transport are domestic duties for millions of households across the globe. People in areas with limited or no access to safely managed sources of water and household energy must fetch these resources on a frequent basis. The health, gender, and economic impacts associated with water and solid fuel collection labor have not been systematically reviewed. (2) Methods: Studies were identified through database searches and included using a list of inclusion and exclusion criteria. Studies were summarized and grouped into one of eight thematic categories. (4) Conclusions: The findings suggest that a diverse and heavy health burden is associated with water and solid fuel collection and transport. The literature also suggests that the provision of safely managed and accessible water and improved fuel options can mitigate these negative outcomes. Filling research gaps and utilizing results to guide policy and funding would likely be an effective way to ensure low- and middle-income countries are not left behind as the world strives to meet the sustainable development goals.

## 1. Introduction

Households in low- and middle-income countries (LMICs), especially rural households, suffer disproportionately from insufficient access to piped water and clean energy [1]. Studies have documented higher rates of disease; missed social, educational, and economic opportunities; and overall lower quality of life associated with this lack of access [2,3]. The Sustainable Development Goals (SDGs), a set of 17 broad goals that aim to achieve a healthier and more sustainable world by 2030, recognize lack of access to safe water and clean energy resources as major global health threats [4,5]. Under each goal, multiple measurable indicators have been established, including criteria that must be met in order to determine whether the broader goal has been accomplished.

SDG indicator 49 aims to track availability of clean water and basic sanitation for all. The World Health Organization (WHO)/United Nations Children’s Fund (UNICEF) Joint Monitoring Programme for Water, Sanitation, and Hygiene (JMP) is responsible for monitoring global progress towards this indicator. According to the most recent data from 2017, nearly 30% of the world still lacks access to safely managed water. Further, only 53% of rural areas have safely managed water, compared to 85% of urban settlements, indicating a significant disparity [6]. A lack of access to safely managed water is a direct threat to the quality and longevity of life, and communities without access are subject to a higher risk of illness and death [7]. Under the water-access indicator, a round-trip to collect water should not take more than 30 min, including queueing time at the source. Thus, water sources that are hard to access due to distance, steep terrain, and other physical barriers that may extend collection time are not considered accessible.

Similarly, an estimated 40% of the world still uses solid fuel for cooking and heating, with rural households more likely to use solid fuel such as wood, charcoal, crop residue than urban households [8,9]. Unlike the water accessibility indicator, there is no SDG indicator for measuring the impact of solid fuel collection labor. Indicator 53 of SDG Goal 7 aims to track access to affordable, reliable, sustainable, and modern energy for all, and it measures the proportion of the world that relies on modern household energy technologies, specifically those using non-solid fuels [10]. Comparable to water collection, long collection times and extensive physical efforts to collect solid fuel are expected to cause similar social, economic, and health problems. As women and girls are often responsible for domestic duties, such as cooking and solid fuel collection, it is reasonable to assume that women and girls are also disproportionately affected by the health impacts of solid fuel collection [2].

This systematic review builds upon previous work conducted by Geere et al. that reviewed the associations between water carriage and water carrier’s health [11]. To our knowledge, this is the first systematic literature review on the human health and development impacts of both water and solid fuel collection and transport. The purpose of this paper is to review and synthesize the evidence of the impacts of water and solid fuel collection and transport on health and human development for households in LMICs. It also aims to identify and describe major themes within the included literature. In the discussion, we identify research gaps and recommend mitigation strategies to make advancements towards achieving SDG Goals and reducing the global disparity in water and solid fuel accessibility.

## 2. Materials and Methods

**Figure 1:**
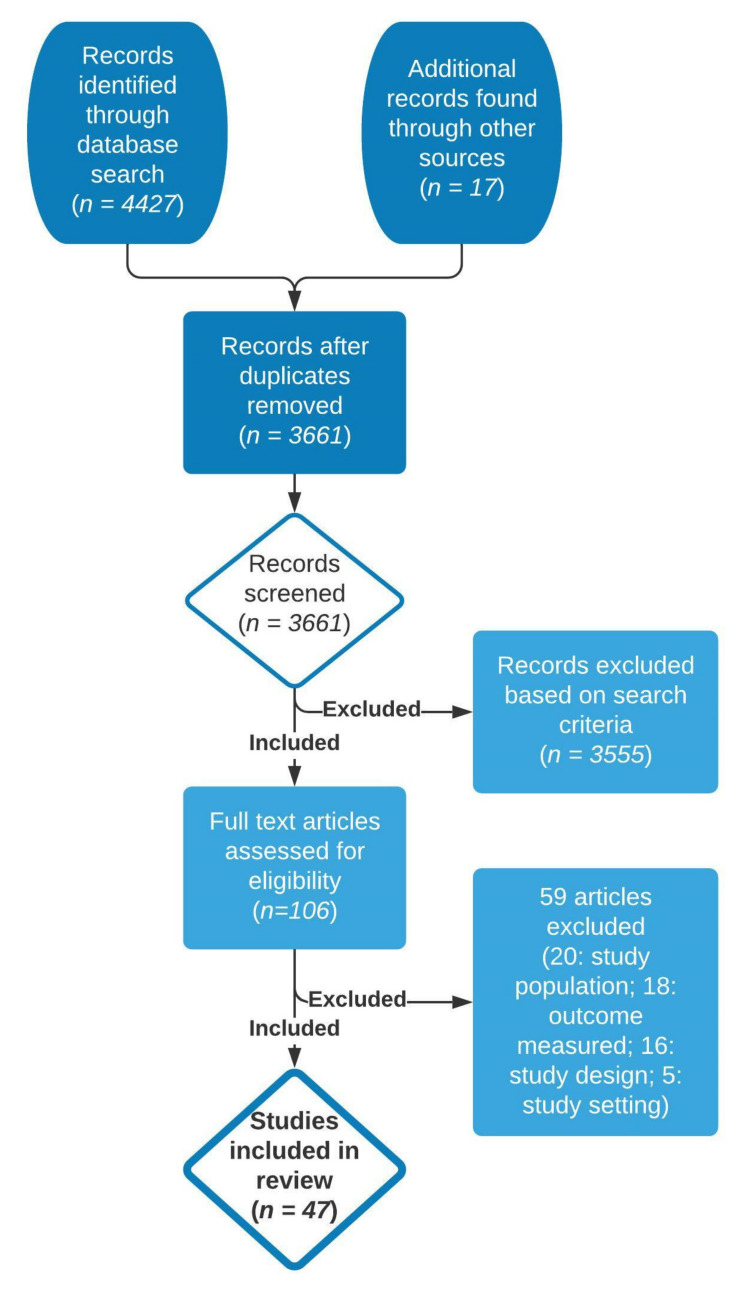
PRISMA flowchart of systematic review process and article screening results.

Boolean search terms were identified and put into PubMed, Embase, and Web of Science databases to collect original research papers. The search terms were selected based on their potential to capture as many water and solid fuel collection and transport articles as possible, while simultaneously narrowing the search to focus specifically on communities in LMICs (see Supplementary Materials Table S1). Additional papers were added after reviewing the citations present in identified water or solid fuel collection and transport studies. The total number of papers screened was 4427 (Figure 1).

The authors used Covidence, a systematic review management software, for four levels of screening. The first level removed duplicates from multiple database searches. The second level required title screening for relevancy, the third level included abstract screening for relevancy, and the fourth level consisted of reading the full text. Each level of screening, aside from the first, required two authors to agree on inclusion of the paper. Studies that reached the fourth screening stage were included in the review if they met a set of inclusion and exclusion criteria. Papers were included if they reported quantitative or qualitative data pertaining to the work of collecting water or solid fuel and its impact on human health outcomes and opportunities for social and economic development. Only studies conducted in LMICs were included. Countries were classified as LMICs based on current World Bank data [12]. Papers written between January 1970 and March 2020 were included. This time frame was selected because of development efforts that started by the United Nations and others to proactively address water accessibility during this decade. Only papers published in peer-reviewed journals and written in English were included. Papers were excluded if they analyzed collection and transport for employment purposes, such as in the case of farmers, porters, water/fuel vendors, or if they analyzed health effects while carrying weights in a controlled environment, such as in a lab or on a treadmill. A total of 47 papers are included in this systematic review: 25 papers were quantitative, 18 were mixed methods, and 4 were qualitative. Additionally, 20 papers utilized surveys, 16 used in-depth interviews with individuals or focus groups, and 8 used quantitative measurement tools, such as odometers or Global Positioning System (GPS) tracking devices. 

**Figure 2:**
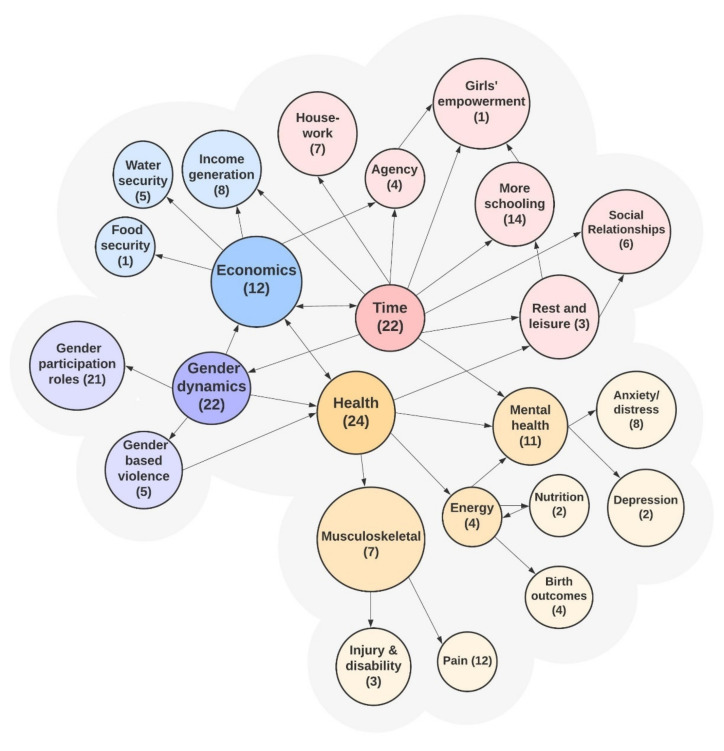
Human health & well-being impacts across different domains described within the literature. The number in each node indicates the number of studies addressing each domain within the review; colors indicate categorization of impacts under broad themes of health, time, gender dynamics, and time with arrows indicating connections between study areas revealed by the literature.

Both qualitative and quantitative results were extracted from all selected papers and summarized (Table A1, Table A2, Table A3 and Table A4), making note of key elements, such as division of labor by gender and/or age of study subjects, and time spent collecting either water or solid fuels. The 47 papers were then sorted into broad impact domains – each with various sub-categories (Figure 2). From the data extractions, the authors identified 8 major themes and summarized respective papers within each theme. 

## 3. Results

### 3.1. Study Characteristics

Of the 4427 studies identified, 47 were included in the final review (Figure 1). Of these studies, 12 assessed the effects of solid fuel carriage. Included studies fell under four major impact domains: (1) health, (2) time, (3) gender dynamics, and (4) economics. Many of the studies were relevant across multiple impact domains, highlighted in Figure 2, which also illustrates the interconnectedness of these impact domains. The impact domains within the figure are categorized by color, with arrows between them indicating connections between study areas revealed by the reviewed studies. Eight reoccurring themes were identified in the review: (1) women’s management of domestic duties, (2) time spent for collection and transport, (3) differences in tools used to collect and transport water and solid fuel, (4) sexual harassment and assault, (5) women and children’s health, (6) psychosocial stress, (7) opportunity costs (e.g., economic, educational, etc.), and (8) lack of autonomy. Below, we describe study findings within each of these eight themes.

#### 3.1.1. Women’s Management of Water and Solid Fuel Collection 

The literature overwhelmingly reflected that the responsibility of water and solid fuel collection, as well as most other household chores, is carried out primarily by women and girls. Differences in gender participation and roles by gender were documented in 21 studies. Singh et al. assessed gender participation in various activities and found that women are responsible for 72% of water collection and 78% of solid fuel collection [11]. Similar trends in gender participation were observed across other studies, with ranges from 54.7–95% of women bearing responsibility in rural areas [2,13,14,15,16]. These differences are often driven by traditional norms and sociocultural stereotypes that deem it shameful, demeaning, and emasculating for men to collect water, especially on a daily basis [14]. Qualitative interviews conducted by Mushavi et al. revealed that a woman can be perceived as a witch or an incompetent caregiver by other community members if her husband contributes to household water collection [17]. Findings illustrating gender disparities as they relate to collection responsibilities and the consequential effects of those responsibilities are summarized in Table A2. 

#### 3.1.2. Time Spent for Water and Solid Fuel Collection and Transport

There were multiple methods used in the studies to measure the time spent on water or fuel collection, which included household surveys to recall the amount of time spent, distance measured by GPS tracking devices, and observations made at water points. 

Several studies were based on data from national Demographic and Health Surveys (DHS) and the Multiple Indicator Cluster Survey (MICS). For water collection time, the DHS includes one main question as a validated measure: “How long does it take for members of your household to go there, get water, and come back?”. The DHS fails to account for how many trips are made. Unlike the DHS, the MICS includes solid fuel collection in addition to water collection measures. Up until 2017, the MICS included one question: “In total, how many hours did (name) spend on fetching water or collecting firewood for household use, since last (day of the week)?”. Recently, the MICS has added a new validated measure to better quantify the number of water collection trips and time spent per trip. As of 2017, the MICS now asks: “How long does it take for members of your household to go there, get water, and come back?” and “Since last (day of the week), how many times has this person collected water?”. The newest MICS version provides the most useful metrics to illuminate the time burden of water collection and transport. Metrics from DHS and/or MICS were used by six studies to assess the time burden [2,3,13,18,19,20]. The new MICS questions have not been implemented in any of the studies included in this review. 

**Figure 3:**
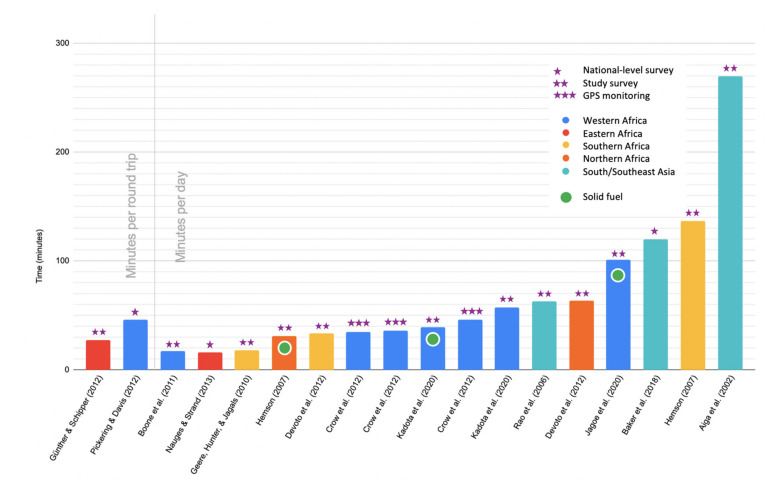
Bar graph shows time spent collecting water and/or solid fuel in studies reviewed. The color of each bar indicates the region where the study took place, the number of stars above each bar indicates the methodology used to produce the estimate, and the bars with a green circle are studies of solid fuel collection while the rest report on water collection.

There was also considerable variability among studies that utilized surveys outside DHS and MICS to measure collection and transport. Some studies applied metrics similar to that of MICS 6 (e.g., round trip time and frequency), while others presented them as daily or weekly averages, and others reported them as time spent per trip—with or without an account of how many trips made daily. Figure 3 shows the time spent collecting and transporting water and/or solid fuel based on the articles included in this review. Many other papers reported ranges or multiple values contingent on some identified contextual state, such as drought or stage in intervention implementation. Many researchers note potential biases in self-reporting time and distances as in national-level or study level surveys. Few, however, deployed GPS-based technologies to gain more complete, accurate data. It is also noteworthy that, as discussed elsewhere in this review, there were substantially fewer sources available with values for time spent collecting solid fuels as compared to water (i.e., three studies assessed solid fuel collection).

Beyond data collection methods and reporting approaches, the literature revealed that differences in time spent were influenced by gender, age, and the remoteness of the community (e.g., urban or rural). Women typically spent much more time engaged in water and solid fuel collection than men. Asaba et al. found that in rural Uganda, men spent only 10–30 min collecting water, while women and youth spent 30–60 min per trip, though all gender groups mostly visited water points once or twice per day [14]. Researchers also found that a greater percentage of children and youth of both genders spent more than one hour collecting water per trip than adults. Time differences between the two groups were attributed to the possibility that children walk slower than adults, are slowed down further when carrying loads too heavy for them, and that they stop to play with other children [14]. Boone et al. observed similar time discrepancies by gender and age in Madagascar, but with the added element of comparing urban and rural communities. In rural areas, on average, women 15 and older spent an estimated 3.3 h per week gathering water; men spent 1.6 h per week; girls spent 2.3 h per week; and boys spent 2.0 h per week. For urban areas, the averages were: 2.0 h per week for women; 1.2 h per week for men; 2.0 h per week for girls; and 1.7 h per week for boys [21]. Additional studies included in this review also reflected the increased time spent associated with living in a rural versus urban area [2,18,20].

Noticeably, these differences in time spent are further exacerbated by changing environmental factors, such as water shortages and deforestation. During dry seasons, water collection times increased substantially. Asaba et al. found that people waited in queues lasting between 2–6 h during these seasons [12]. With regard to solid fuel collection, Jagger and Perez-Heydrich found that deforestation resulted in increases in the time spent collecting wood fuel by 33.1%, as it took longer to collect fuel in woody savannahs and degraded forests than healthier forest ecosystems [22].

Overall, the literature indicated that the time burden associated with water and solid fuel collection varies by gender, age, rurality of the community, and environmental factors.

#### 3.1.3. Differences in Tools Used to Collect and Transport Water and Solid Fuel

Twenty of the studies presented information on the methods used to collect and transport water or solid fuel. Head-loading and back carriage were the most frequently documented methods of transporting both water and solid fuel in this review, though use of wheelbarrows, bikes, rolled drums, and donkey carts were also observed in the literature. In one population of 39 South African water carriers, Geere and Hunter observed that 30 people used head-loading, two rolled a water drum, and seven pushed a wheelbarrow [23]. Another study also conducted by Geere et al. within rural South Africa observed that 53% of children used head-loading, 10% used a wheelbarrow, 10% rolled a drum, and 21% used donkey carts [24]. 

There was also variability in transport technology use by gender. Robson et al. found that boys who participated in water collection had greater access to technologies that aim to mitigate head loading and back carriage versus girls [25]. While headloading is almost ubiquitous for girls, boys more commonly reported hand-carrying loads or using carts or bicycles. A study in Haiti tested whether backpacks designed to collect and transport water would reduce the physical impacts of head loading; the researchers found that women were less likely to use this intervention on a sustained basis versus men [26]. Ferguson found that in a rural agricultural community in Kenya, only 13% of women used collection and transport technologies compared to 58% of men [15]. Similar observations were by made Porter et al. in South Africa, where 40% of girls and 23% of boys reported head-loading water on their most recent journey, while 35% of girls and 44% of boys used carts or bicycles [27]. Qualitative interviews from the same study suggested that brothers of female water carriers usually had priority access to any available technology for transporting water [27].

#### 3.1.4. Sexual Harassment and Assault

Seven of the studies documented that individuals, mostly females, felt scared or unsafe when travelling to collect water or solid fuel, as community-level social conditions placed them at a greater risk of sexual harassment and assault along their journeys. This was more evident when distances were greater and when travel occurred during early morning hours or at night. Six studies included qualitative interviews in which women reported occurrences of verbal abuse, rape, and attempted assault against them and younger girls. Ayoade and Sikiru found that out of 800 girls interviewed in Nigeria, 57% reported being sexually harassed and/or assaulted while travelling to a collection point [28]. In addition to sexual harassment, cases of water vendors forcing women and girls to have sex with them in exchange for access to water have also been found to be a common phenomenon in some areas [29]. The findings of six relevant studies as they relate to gender-based violence are shown in Table A3.

#### 3.1.5. Women and Children’s Health

The health effects of repeatedly carrying heavy loads were frequently documented. A total of 27 studies assessed associations between the need to collect water and/or solid fuel and poorer health in women and children, with varying impacts as they relate to both physical and mental health. The studies reviewed identified multiple physical health effects that result from the physiological burden of both water and solid fuel transport, including: headaches, chest pain, joint pain, neck pain, stiffness, fractured bones, nasal bleeding, as well as accidental injuries. The findings of 15 studies pertaining to the physical effects of water and fuel carriage are presented in Table A1, and among those, self-reported pain was studied and documented in 12 studies, making it the most frequently reported indicator of health impacts. We assumed the quality of self-reported data pertaining to perceived pain in these studies to be valid, as pain is a subjective and emotional experience [30]. However, it should be mentioned that Porter et al. discussed the possibility that girls underreport their pain because water/fuel collection is expected of them [27]. In a study conducted on water carriers in Limpopo Province, South Africa, Geere et al. found that 69% of participants reported spinal pain and 38% reported back pain due to water-carrying, with head loaders reporting musculoskeletal pain more frequently than water collectors who used other carrying methods. Additionally, this study identified several other musculoskeletal structure risks that water-carriers face, such as spinal fractures, spinal dislocation, and even death [31]. A cross-sectional study in South Africa, Ghana and Vietnam assessed water carrying methods and health status [32]. The research found that those who reported recent collection and transport of water were at a significant risk of reported pain associated with spinal axial compression in the cervical region. The authors highlighted that musculoskeletal diseases are a common cause of disability in LMICs and that water collection and transport likely plays a major role [32].

Complaints of high physical energy demands and fatigue were also reported in four studies. In an assessment of the physiological burden associated with water carriage in terms of kilocalorie expenditure, researchers found that the drawing of water and return journey was “very heavy” for younger age groups and “heavy” for older age groups [33]. Rao et al. came to similar conclusions, finding that carrying two water containers on the head was one of the most tiring activities and required more energy than actually drawing water from the well or using the hand pump [34]. 

#### 3.1.6. Psychosocial Stress

In addition to physical health impacts, evidence of worsened mental health and psychosocial stress were documented in 11 studies. Here, we use the term psychosocial stress as an umbrella term to encompass depression, stress, anxiety, and fear. In a mixed-methods study exploring the relationship between water insecurity and depression in rural Uganda, Mushavi et al. found that water insecurity led to emotional distress and feelings of “choicelessness”, that subsequently resulted in negative impacts on social behavior, relationships, and cultural perceptions of gender roles [17]. However, the study observed that the severity of depression was greater among men than women. A second study also conducted by Cooper-Vince et al. in rural Uganda, it was found that household water insecurity led to caregiver depression, which acted as a mediator variable underlying the causal relationship between water insecurity and children missing school [35]. Furthermore, a study conducted in a rural agricultural community in Kenya indicated that women in particular endured high levels of stress and poorer health due to eco-demographic pressures (e.g., water scarcity) placed on the women in such communities [15].

In a cross-sectional study evaluating water access-related emotional stress, Thomas and Godfrey found that emotional distress was not significantly associated with accessibility of the main water source and time taken for collection. Rather, the primary concern of collectors was collection during the night or very early in the morning [36]. This is likely because the act of collecting water and solid fuel can jeopardize collectors’ physical safety by exposing them to greater risk of physical or sexual assault and animal attacks. In a separate study, Robson et al. administered questionnaires which revealed that 22% of children reported encountering hazards (dangerous animals, vehicles, crossing rivers, harassment, violence) when collecting water [25].

Krumdieck et al. found that 77.3% of their study participants felt “somewhat or strongly concerned” for their physical safety when making trips to water collection points [16]. Henley et al. tested the association between hair cortisol content–as an indicator of chronic stress–and various cultural and socio-economic conditions by which researchers found that almost a quarter of sub-Saharan water collectors did not feel safe when collecting water and also had a higher average hair cortisol content (639 ± 300 ng/g) compared to a Caucasian reference group (299 ± 110 ng/g; one-way analysis of variance (ANOVA); *p* = 0.0003) [37]. Fourteen participants surveyed reported having ever been assaulted during water collection, although no difference was found in cortisol content between those who had or had not been assaulted, suggesting that it is the fear of attack that contributes to higher stress levels [37]. 

The burden of water collection can also translate to a strain on families as a whole [38]. Primary water collectors are often also the primary child caretakers, and being gone for extended periods of time may result in irregular meal times and food availability, inability to converse with family members regarding household- or school-related issues, lack of water for bathing and cleanliness. The authors suggested that all of these spillover effects can be manifested as stress or discontentment among household members. Although water-access-related stressors were found to contribute to psychosocial stress through the previously described mechanisms, there were other stressors experienced by communities that were ranked higher in importance. Stevenson et al. found that issues related to illness, death, and poverty were of greater concern [39]. 

#### 3.1.7. Opportunity Costs

One of the major impacts associated with collection and transport tasks was reduced opportunity to be involved in educational, economic and leisure activities. The time spent on collection and transport of these essential resources was time diverted from other desirable activities, with varying subsequent impacts on wellbeing. One of the most commonly reported opportunity costs was education, with fourteen studies citing evidence of negative impacts on schooling or positive impacts after an intervention that improved access. Ayoade and Sikuru found that out of 800 young girls surveyed in Nigeria, 97% reported ever being late to school and 86% reported poor school attendance as a result of water collection duties [28]. 

Negative impacts on income generation were also found in eight studies, with data showing collection duties resulting in being late or missing paid work entirely. Subbaraman et al. found that 58.7% of water collectors reported negative impacts on work as issues with access and reliability force them to miss days of work entirely or leave jobs early to procure water [40]. 

The literature revealed that time spent on water and solid fuel collection also resulted in a multitude of other missed opportunities, the effects of which could be alleviated through potential or already implemented intervention strategies. Table A4 presents reported effects of the time burden of load carriage on housework (including childcare), rest and leisure, school attendance, income generation, and health from studies that focused, at least in part, specifically on opportunity costs. Although the time burden associated with water collection compared to fuel collection was more frequently investigated within the literature, the effects of the time burden are assumed to be comparable.

#### 3.1.8. Lack of Autonomy

The literature revealed that women often lack autonomy and power within their communities to make decisions around water collection and transport due to gender norms and unequal power relations [41]. No studies were identified that presented data on autonomy in the context of fuel collection, only water collection. Yerian et al. observed that despite women bearing the primary responsibility in water collection, women are typically not present at water management committee meetings or merely hold “token membership” and are therefore excluded from decision-making processes that could mitigate the burden they experience [42]. In assessing the role of women in water management and conflicts, the Varickanickal et al. revealed that although in some areas both women and men believed women to have a valuable role to play in water management, cultural norms and restrictive definitions of appropriate female behavior deemed it disrespectful for women to speak in front of men; so, if women do attempt to contribute, their concerns are unlikely to be acknowledged. The authors also found that female participants discussed barriers to their efforts to improve access to water services [41]. The women stated how unequal power relations limit their ability and capacity to challenge the government’s inaction toward service provision. When making appeals to government authorities for improved access to water, research found that those in power typically remained unresponsive to requests as their inaction goes without consequence [41]. Although some women’s community organizations have found ways to solve conflicts outside of established committees, women also described trust issues that prevented community mobilization efforts to implement change [41,42]. There was general consensus in the studies reviewed that women should have greater participation in water management decisions. However, guidance on how to implement more meaningful involvement were missing from many of the studies.

## 4. Discussion

Based on the studies included in this review, sufficient evidence exists to demonstrate the wide-ranging impacts of water and solid fuel collection and transport on health and overall quality of life among women and children in LMICs.

The papers regarding women taking on leadership roles for household duties reveal that there is a great societal burden placed on women to perform their domestic chores well. Rather paradoxically, leadership in household duties does not always equate to autonomy and empowerment. Various studies included in this paper show that women have higher levels of stress because of sociocultural expectations for them to run a household and collect water and/or solid fuel with efficiency [15,17,41]. Yerian et al. revealed that although women are the primary water collectors, they are not allowed to participate in water management committee meetings, and instead must find other ways to empower themselves [42]. Other studies have shown that policies and government orders are not sufficient in overcoming deeply-rooted sociocultural barriers [43]. However, it is possible to mitigate these barriers through providing better education opportunities and government enforcement of placing women in decision-making processes [43,44].

In the papers analyzing time spent on collection and transport activities, most found that women and children are the primary actors. Multiple studies show that women, girls, and people living in rural settlements are more likely to spend longer amounts of time collecting water and solid fuel than their male or urban counterparts, taking away time that could be used for rest and education [2,18,20,45]. Therefore, more time allocated to collecting water and solid fuel negatively impacts women and girls by barring them from enrichment opportunities and depriving them of time to take care of their mental health by resting or having the freedom of leisure time. 

Additionally, when a large amount of time is spent on collecting either fuel or water, women are less likely to be able to care for young children properly. Geere and Hunter, highlighted that young children in households where women are the primary collectors are more likely to be left unattended for over an hour [23]. In households where children are the primary collectors, children are at a higher risk of contracting a diarrheal disease and encountering other dangers such as wild animals, hazardous terrain, and assault [3,25,31]. 

With worsening deforestation and global climate change, communities with already limited access to water and solid fuel may be finding themselves travelling longer distances and taking more time to collect their needed resources. If collecting resources becomes impossible, the livelihoods of people in vulnerable communities will be significantly impacted by negative health outcomes and increased time demands of water and fuel procurement [46]. As multiple studies noted, people in resource-scarce communities make multiple trips to collect water and fuel numerous times a week, and the frequency with which they have to perform this task will only increase in response to environmental changes. Thus, providing accessible, safely managed water and fuel is crucial to ensuring preservation of these communities and improving their health and development.

Another theme that highlighted the sociocultural gender imbalances was the variety of tools used to collect resources. As shown by Robson et al., women and girls are more likely to carry heavy loads on their heads, while men and boys are given more advanced tools, such as bicycles or wheelbarrows to transport loads. Though load carrying is a physically demanding chore overall regardless of technologies used, head loading subjects females to a higher risk of chronic pain and injury to their musculoskeletal structures [25,31,47]. Additionally, the use of bicycles and cars to collect water and fuel may take less time than head loading or rolling water drums, which are done on foot. However, women are frequently prevented from using these technologies because these vehicles are typically regarded as men’s property [48]. Thus, those who have the privilege of using more advanced transportation technologies suffer less from time burdens and have a lower risk of injury or fatigue. Research has also suggested that men’s involvement in roles traditionally seen as ‘women’s’ work increases when it is made less physically tiring by transportation or other technical intervention [49]. Education to dismantle gender restrictions on vehicle use, and the provision of more modern transportation tools to reduce human energy expenditure and improve time management will likely be beneficial to groups who still collect water using more rudimentary methods that have been shown to cause injury and irritation primarily to the neck, head, and hands.

Women in communities with poor water and solid fuel accessibility are also at higher risk of being sexually harassed or assaulted en route to collection sites [6,25,50,51]. The threat of being attacked becomes a significant cause for anxiety and distress, especially for women and girls [52]. One study which involved key informant interviews with women found that the predictable routes women take to collection sites, long travel times and distances, and the fact that women tend to travel early in the morning or late at night to avoid long queues leaves them vulnerable to gender-based violence [42]. If resources are readily available on premises and in alignment with SDG goals 6 and 7, women can avoid being forced to navigate unsafe conditions and lower their risk of gender-based violence and the subsequent associated anxieties and worries. Remedying these issues will require intense education interventions and strict persecution of perpetrators in addition to the provision of safely managed, and safely accessible, water and fuel sources.

Inaccessible water and solid fuel sources have a direct negative effect on human health, and women and children are disproportionately affected, as demonstrated in multiple papers and throughout this review. Common health problems mentioned in these papers include headaches, neck pain, hand pain, and fatigue [24,53,54]. Additionally, children in Hemson’s study were able to associate their tiredness, illness, and pain with their water collection duties [54]. Head-loading is also associated with head, neck, and back pain, and there are higher rates of musculoskeletal pain among head-loaders compared to people who utilize other methods. As mentioned previously, women and girls are more likely to head load, thus making them more likely to suffer from chronic musculoskeletal pain compared to males. Those responsible for collecting water are also more likely to contract waterborne diseases because they are in closer and prolonged contact with untreated water, and a five-minute decrease in water collection time is associated with lowered risk of contracting a diarrheal disease [3]. Reducing the time and distance needed to access water and ensuring water sources are safely managed and contaminant-free can successfully reduce the risk of transport-related injury and diseases in those primarily responsible for water collect, women and children. It is reasonable to assume that common health risks associated with the journey required to collect water previously mentioned, such as dangerous terrain, wild animals, and musculoskeletal pain also apply to solid fuel collecting [27,55]. Thus, the provision of safely managed water on premises and modern fuel technologies will reduce dependence on women and children to collect enough resources for their households and mitigate the unique disparities women and children face.

As holistic health involves mental as well as physical health, psychosocial stress is a crucial theme to understand the true scope of health impacts associated with water and solid fuel collection. Communities facing resource scarcity are more likely to suffer from depression and emotional stress [17]. In the work of Stevenson et al., study participants acknowledged that insufficient water, using untreated water, and missing opportunities for other tasks and activities were sources of stress [39]. Some participants also reported having to steal water from others. Resource scarcity has the potential to deeply affect social relationships and negatively impact mental health. Additionally, as seen in the sexual harassment section, women and girls experience emotional distress at the threat of violence against them while they travel to collect water or fuel. As women are largely responsible for resource collection, they are again disproportionately affected by stress, anxiety, and depression compared to men [56]. Mental health conditions of people in underserved and rural communities may also be underdiagnosed or neglected due to sociocultural misconceptions of mental illnesses [57]. In addition to the need for more mental health support services, more understanding and awareness of mental health issues, research is needed on the mental health impacts of water and fuel collection and more work should be conducted to develop appropriate interventions that would positively influence mental health.

Among the studies that discussed opportunity costs, the major opportunities lost were related to education, rest, and economic development. Women and children, specifically girls, lose more opportunities for social and economic growth because of the large amount of time and energy water and fuel collection demands. More girls than boys miss school because of water collection, and as children grow older, girls are more likely to drop out of school to continue with household duties [18,58]. Additionally, children responsible for collecting water or fuel in the morning may be tardy, too tired to perform well at school, or lack time needed to do schoolwork [24,27]. In terms of economic opportunity, Jagoe et al. found that women reported more time to be economically productive when they were provided with modern cooking technologies, resulting in financial stability and independence [59]. Further, in the work of Aiga et al., households with improved water sources were able to generate higher incomes by using time saved for income-generating tasks [19]. Therefore, improved resource accessibility can also improve the economics of individuals, as well as entire settlements, which can alleviate stress and dependence on the community members in charge of resource collection, commonly women and children [19].

This systematic review has identified several research gaps. First, there is a significant difference in volume of literature investigating water collection and transport compared to solid fuel, indicating a lack of research evaluating solid fuel carriage and the associated impacts on human health. There is likely a significant overlap between the effects of both water and solid fuel carriage, particularly when focusing on distance traveled and time spent. Nevertheless, solid fuel carriage and the associated health risks are still unique and require equally innovative and unique solutions. Further investigation of the burdens of solid fuel collection and load carriage is essential to understand the broader burden of load carriage, and to offer solutions that alleviate negative health impacts. 

An important limitation of existing studies is the lack of standardization in measurement in cross-sectional and survey-based studies. In the absence of validated measures of water and solid fuel collection, comparing data from various sources, such as DHS and MICS surveys, can be difficult and unreliable. Additionally, as seen in Figure 3, researchers have used national-level survey data, study survey data, and GPS monitoring for collecting time data, indicating a lack of standardization in methodologies. As survey data largely rely on self-reporting, there is risk of biased, inaccurate reporting. Assuming cost and feasibility concerns are appropriately managed, researchers may be able to reduce measurement error by utilizing GPS-tracking or direct observation [44]. A study conducted by Davies et al. tested several different GPS-tracking devices on several different factors, including indestructibility, battery life, and price [60]. The model the authors found to be the best was also used by them in a study tracking water collection time and distances in Kenyan informal settlements [45]. Additional cost–benefit analysis of GPS-tracking technologies versus direct observation/self-reporting may be an area of interest to future researchers. 

Finally, many studies described the negative impacts load carriage has on livelihoods and proposed appropriate interventions, but few studies actually evaluated interventions that set out to reduce this burden. Studies that can implement and test interventions to improve access to safely managed water and household energy and evaluate the health and development effects will expand the scope of knowledge on these topics. These studies can also guide future policy measures and plans to create sustainable, long-term solutions. With more attention given to water- and solid fuel-related issues, there may be more opportunities for funding and governmental support to mitigate these problems and improve the health outcomes and livelihoods of communities around the globe.

## 5. Conclusions

The evidence collected in this systematic review suggests that improved access to safe water supply and modern fuels and technologies have the potential to positively impact the livelihoods of millions of people. Further investigation of the burdens of water and solid fuel collection and load carriage is essential to understand the burden of these activities and offer solutions that could mitigate these impacts. Additional rigorous studies that analyze health and development impacts in relation to interventions are needed. Identifying the problem is only the first step in improving health and development, and new bodies of evidence based on efforts to mitigate the problem are needed. Based on the results of this study, we have listed several recommended next steps to improve research in this particular field. 

Global climate change: Global climate change is likely to be a major influencer on water and fuel resource availability and access issues could potentially worsen. Research studies are needed to better characterize the association between environmental changes and water and solid fuel collection and transport. Again, studies are needed that test interventions that aim to mitigate water scarcity during dry seasons or assess the feasibility of using new energy and technologies to provide access to modern cooking and heating.

Technology and service innovations: Technological and service upgrades, such as providing piped water and access to clean fuels, will likely mitigate water and solid fuel collection. This in turn will reduce the risk of associated detrimental impacts, such as chronic musculoskeletal injury and time poverty. Advanced technologies and improved services have the potential to also reduce dependence on solid fuels, which can reduce health risks associated with household air pollution and simultaneously decrease the need to forage for fuels, which may become increasingly difficult as urbanization and deforestation increase. As mentioned by many studies, women and children are disproportionately affected by the water and fuel transport and also suffer greater exposures to household air pollution. Additionally, there may be short-term solutions that create more ergonomically advantageous alternatives to facilitate transport of water and solid fuel. These alternatives, however, should be rigorously tested with key stakeholder involvement.

Standardization of data collection: As stated previously, significant variability exists in the measurement of water and solid fuel collection and transport exists across DHS, MICS, and other methods of data collection used in different countries. In order to address this issue, specific indicators pertaining to water and solid fuel collection and transport need to be defined and measured at sub-national levels in order that progress towards SDGs and local goals can be measured. Indicators measured by MICS, DHS, and other surveys must be improved and standardized to accurately track and end the practice of water and solid fuel collection and transport.

Current research exists suggesting that there are improved water security metrics. The recently developed 12-item Household Water Insecurity Experiences (HWISE) Scale measures universal experiences of household water insecurity across LMIC countries using simply worded questions related to household water access, availability, and use, and can be administered in approximately four minutes. This provides the ability to better quantify the prevalence, causes and consequences of household water insecurity, and can contribute to an evidence base for clinical, public health and policy recommendations regarding water [61]. Additionally, a reduced four-item HWISE Scale has also been shown to be a valid tool for assessing water issues related to health and well-being when resources are constrained [62]. 

The majority of studies in this review focused on water and most used national survey data; only three used GPS monitoring (Figure 3). Surveys are potentially prone to bias and depend largely on how well participants can recall the time and distance of their collection routes. Therefore, other means, such as GPS monitoring, if feasible, may be the most objective method of measurement. Better measurement data are likely to be more effective in advocating that policymakers, and other stakeholders, consider the true burdens of time poverty and long distances that water and solid fuel collectors face.

Interlinked human health: As discussed in the introduction, the two SDG goals in focus are goals 6 and 7, as they pertain to water and household energy. However, there are additional SDG goals that may be met upon providing safely managed water and modern fuel to poor households. Improvements would likely contribute to SDG 3, which aims to ensure healthy lives and promote well-being for all ages, although these gains need to be more completely documented across multiple contexts. Due to the negative impacts on school attendance and income generating activities, reduction of time spent collecting and transporting these resources may also contribute to SDGs 4 and 8, which emphasize quality education, decent work, and economic growth opportunities for all. The causal pathways linking load carriage to these outcomes, however, can be complex and context-specific, so additional studies are needed to fully characterize and scale these outcomes. SDG 5, which aims to empower women and girls and ensure their rights will also potentially be affected by improving access to water and household energy. 

The review overwhelmingly revealed that water and solid fuel collection are considered a woman’s responsibility and that women are prevented from having power to make decisions regarding water and fuel management. This evidence suggests that by increasing gender equity and female participation in water management practices, communities may begin to dismantle these detrimental gender norms, cultural expectations, and unequal power relationships that contribute to the disproportionate health impacts associated with collection duties experienced by women and children. Studies focusing on exploring the impact of various water management policy reforms on gender dynamics could identify critical success factors for relevant populations.

## Data Availability

All articles and reports used for this review are available to the public.

## Supplementary Materials

The following are available online at https://www.mdpi.com/article/10.3390/ijerph181910355/s1, Table S1: List of Boolean search terms.

## Appendix A

**Table A1:** Physical impacts associated with water and fuel collection and transport.

Author	Population	Water or Solid Fuel	Experimental Design	Time Frame	Carrying Method	Load Weight	Findings
Asaba et al. (2013) [14]	Rural Uganda: 602 HHs	Water	Mixed Methods	Apr. 2011–Jan. 2012	Head loading	3–20L jerry cans	Carrying water was perceived to cause chest pain (33%–64%); headache (5%–23%); nasal bleeding (0.8%–4.0%); back pain (0.8%–1.9%); and spinal problems (0.3%–0.8%).
Ayoade & Sikuru (2015) [28]	Peri-urban Nigeria: 827 children (ages 5–15)	Water	Qualitative Survey	Nov. 2013–Feb. 2014	Head loading	25 L Jerrycans	788 girls (95%) experienced neck/back pain from excessive loads; most reported a belief that their back pains worsened during menstruation as a result of heavy water carrying.
Ruplekha et al. (2009) [33]	Rural Nigeria: 30 individuals	Water	Observational	ND	Handheld buckets	Avg. 10.60 kg	Drawing of water and return journey was found to be very heavy and heavy for younger and older age groups, respectively; musculoskeletal problems identified in older age groups.
Buor (2004) [63]	Urban Ghana: 212 women	Water	Cross-sectional survey	2001	ND	ND	39.2% of women spent > 4 h/day collecting water were sick once every 2 weeks compared to 21.3% of women who spent less than 2 h/day.
Geere, et al. (2018) [32]	Ghana, South Africa, Vietnam: 997 HH	Water	Cross-sectional survey	Jun.–Dec. 2012	Head loading	ND	Water carriers more frequently reported pain associated with spinal axial compression in the cervical region; head loading likely to be a major contributing factor in musculoskeletal diseases.
Geere & Hunter (2010) [31]	Rural South Africa: 39 water carriers	Water	Mixed methods	Mar.–Oct. 2008	Head loading	~41% of body wt.	69% reported spinal pain and 38% reported back pain associated with water transport.
Geere et al. (2010) [24]	Rural South Africa: 32 Children	Water	Mixed methods	ND	Head loading, wheelbarrow, rolled drum, donkey carts	ND	Children perceived that water collection and transport caused hand and joint pain, tiredness, and injury.
Hemson (2007) [54]	Rural South Africa: 2053 children	Both	Cross-sectional Observational Survey	ND	Head loading, wheelbarrow	~25 kg	96% said carrying water is tiring, 75% reported fatigue, and 96% reported neck pain; 57% sought medical attention.
Kadota et al. (2020) [64]	Rural Tanzania: 82 women (≥18 yo)	Both	Cross-sectional	July–Aug. 2016	Head loading	Avg. 18.8 kg	65% reported lower back pain that lasts >3 days; 66% reported neck pain; Odds of having lower back pain increased 10% with every additional 1kg load increase.
Matinga et al. (2013) [65]	Rural South Africa: 10 nurses servicing ~20 villages	Fuel	Mixed methods	2007, 2009	Head loading	Avg. 70kg	Nurses stated that women reported thoracic cavity stress, neck pain and stiffness, fractured bones, and superficial wounds from collecting firewood.
Porter et al. (2012) [27]	Ghana, Malawi, South Africa: 2967 children	Both	Cross-sectional	2006–2009	Head loading	ND	4.6% of children complained of tiredness and 66% reported chronic head, neck, back, and waist pain. Study suggested that girls ay underreport pain because water/fuel collecting is their duty.
Rao et al. (2008) [34]	Rural India: 22 individuals	Water	Cross-sectional	ND	Head loading	~25 kg	Carrying two water containers on the head was one of the most tiring activities and required more energy than actually drawing water from the well or using a hand pump.
Robson et al. (2013) [25]	Malawi: 1504 children	Water	Mixed methods	ND	Head loading	10–19 kg	35% of children reported pain and difficulty carrying water; 26% reported head and neck pain. Other issues reported were pain in other areas, difficulty breathing, and tiredness.
Singh et al. (2012) [53]	Rural India: 100 agricultural workers	Both	Cross-sectional survey		head loading (females); head/shoulder carrying (males)	Fuel wood: 30–45 kg	Respondents reported severe neck and shoulder pain during water collecting; severe lower back pain felt by female respondents during water collecting.
Subbaraman et al. (2015) [40]	Urban India: 6 focus groups, 40 in-depth interviews, 521 HH surveys	Water	Mixed methods	ND	ND	ND	Water collectors, especially the elderly, expressed concerns about the physical challenges of water collection.

**Table A2:** Gender disparities in water and solid fuel collection and transport.

Study	Population	Water or Solid Fuel	Experimental Design	Time Frame	Findings
Asaba et al. (2013) [14]	Rural Uganda: 602 HHs	Water	Mixed methods	Apr. 2011–Jan. 2012	54.7% said that women collected water in their HH, followed by female children (19.4%); adult males (9.4%); female youths (5.7%); male children (5.6%), male youths (3.5%) and HH helps. 90% of females head load, only 37.6% of males do. Pain reported more frequently by women and female youths.
Ayoade & Sikuru (2015) [28]	Nigeria: 827 children	Water	Qualitative	Nov. 2013–Feb. 2014	The results indicated that the ratio of female to male was thirty-to-one (30:1) in household water provision.
Baker et al. (2018) [20]	India: 7926 women	Water	Cohort	2004–2005, 2011–2012	Time to source, type of source, and time spent collecting were not associated with pre-term births (PTB); Low birth weight (LBW) was associated with spending >2hrs collecting, but not with type of water source, or time to source; Harassment of women is associated with PTB and LBW.
Bisung & Elliot (2017) [56]	Kenya: 557 HH	Water	Cross-sectional	July 2016	Female-led households reported 1.15 points less on the Household Water Insecurity Access Scale than male-led households. (OR = 1.85. 95CI: 1.1 – 3.1).
Boone et al. (2011) [21]	Madagascar: 2190 HH	Water	Descriptive statistical analysis	2004–2005	Women comprise the majority of water collectors; In rural areas, women spend on average 3.3 h versus men who spend an average of 1.6 h.
Cooper-Vince et al. (2017) [35]	Uganda:257 women and 552 children	Water	Cross-sectional	ND	44% of female HH heads scored above the 1.75 point cutoff, indicating possible depression; HH water insecurity is associated with children missing school; 16% of this association is mediated by mothers/female caregivers having depression.
Crow et al. (2013) [45]	Kenya: 50 HH	Water	Cross-sectional and spatial analysis	Summer 2010, fall 2011	Women suffer more from time poverty than men do because they are primarily responsible for water collection.
Mdaghri (1995) [66]	Morocco: 156 HH	Both	Case study	ND	Women are responsible for the majority of work that is impacted by environmental degradation, causing them to feel burdened; Environmental problems and community-level social barriers make it difficult for women in these villages to thrive.
Ferguson (1986) [15]	Kenya: 524 HH	Water	Mixed methods	Oct.–Nov. 1983	~70% of water collection is performed by women; 87% of women do not use transportation technologies compared to 42% of men; 4.5% of men between the ages of 20–29 were disabled, while 9.7% of women 20–29 were disabled.
Geere & Cortobius (2017) [13]	Multiple countries: 371635 HH	Both	Synthesis of survey data	2010–2015	Women make up 60.88% and 46.26% of water collection duties in rural and urban areas, respectively; In HH where women collect water, women are less likely to utilize a healthcare facility during childbirth, less likely to utilize antenatal care, and children under the age of 5 are more likely to be left at home alone for an extended period of time.
Geere & Hunter (2020) [23]	Multiple countries: 2740855 individuals	Water	Cross-sectional	2009–2014	Women who need to collect water have reduced odds of giving birth in a health care facility and reduced utilization of antenatal care. Children in homes where women are water collectors are more likely to spend long periods of time unsupervised.
Graham et al. (2016) [2]	24 countries: DHS/MICS survey data	Water	Synthesis of survey data	2005–2012	62% of girls collect water compared to 38% of boys; Adult females are primary water collection laborers in more than 75% of households.
Henley et al. (2014) [37]	Kenya: 108 individuals	Water	Cross-sectional	Jun.–Aug. 2011	Increased hair cortisol levels found in females when compared with males from the settlement communities.
Krumdieck et al. (2016) [16]	Kenya: 323 pregnant women	Water	Observational cohort	Sep. 2014–Jun. 2015	Women bore the primary responsibility of acquiring water in 77.5% of HH, even while in late pregnancy (mean (SD) gestational age of 33.1 (2.1) weeks).
Mukuhlani & Nyamupingidza (2014) [67]	Zimbabwe	Water	Qualitative case study	ND	Water scarcity has increased the productive role and responsibility of women to collect water. However, it was observed that youths between the ages of 18–25 were going to collect water without much gender disparity, explained by the fact that most of them were school leavers.
Mushavi et al. (2020) [17]	Uganda: 1642 adults	Water	Mixed methods	ND	Women experienced severe emotional distress when water is unavailable; the association between water insecurity and depression was greater for men than women.
Nauges & Strand (2013) [18]	Ghana: 405 clusters of HH	Water	Descriptive statistical analysis	1993–1994, 1998–1999, 2003, 2008	Longer water hauling time lowers the proportion of 5-15 year old girls attending school. Girls that take >20 min are less likely to attend school. HHs with female heads increases proportion of girls at school.
Porter et al. (2012) [27]	Ghana, Malawi, South Africa: 2967 children	Both	Cross-sectional	2006–2009	In Ghana, 95.9% of girls in remote rural settlements collect water every day compared to 52% of urban girls; In Malawi, 71.5% of all girls carry water while 29.8% of all boys carry water; 40% of girls headload compared to 23% of boys; Boys are more likely to use their hands or push carts. 35% of girls and 44% of boys used bikes.
Robson et al. (2013) [25]	Malawi: 1504 children	Water	Mixed methods	Oct. 2006–Mar. 2009	Boys who participate in water collection have the assistance of technologies but girls rarely do; 89% of girls and 66% of boys carried water overall; 68% of girls and 32% of boys carrying daily; 10%of girls and 6% of boys missed school due to water carriage.
Singh et al. (2012) [53]	India: 100 agricultural workers	Water	Cross-sectional	ND	Males brought water from nearby sources (<2 km); females walked longer distances; Females had much greater participation % in collecting water for HH purposes and fuel wood carrying.
Stevenson et al. (2012) [39]	Ethiopia: 39 women	Water	Mixed methods	Nov.–Dec. 2009, Feb. 2010	Water insecurity is associated with psychosocial distress among women; In 54% of HH, women and children collect water. 93% of women made decisions about where to collect water and 98% decide how much to collect without consulting their husbands/other kin. Other HH chores do not see major gender variation.
Varickanickal et al. (2020) [41]	Kenya: 13 women	Water	Photo voice	Jul. 2016	Women and girls, as the main water collectors, share similar concerns regarding their health and safety related to water collection. However, they lack autonomy and power within the community to make changes due to gender norms and unequal power relations.
Yerian et al. (2014) [42]	Kenya	Water	Mixed Methods	May–Aug. 2011	Though women are heavily involved in water collection, they are not present at water management committee meetings nor part of the decision-making process; Culturally, women are expected to remain quiet in front of men, and if they do speak, they are ignored.

**Table A3:** Gender-based violence associated with water and solid fuel collection and transport.

Study	Population	Study Type	Dates	Findings
Asaba et al. (2013) [14]	Rural Uganda: 602 HH	Mixed Methods	Apr. 2011– Jan. 2012	Reports of rape and attempted assault against women and children. Instances of verbal attacks, especially insults towards girls.
Ayoade & Sikuru (2015) [28]	Peri-urban Nigeria: 827 children (ages 5–15)	Qualitative Survey	Nov. 2013–Feb. 2014	Out of 800 girls interviewed 57% reported being sexually harassed and/or assaulted during water collection.
Baker et al. (2018) [20]	India: 7926 women (ages 15–49)	Cohort	2004–2012	Community-level social conditions related to water collection (i.e. becoming a target for gender-based harassment/violence while walking to a drinking water source) make pregnant women more vulnerable to pre-term birth and low-birth weight.
KEWASNET and ANEW (2020) [29]	Kenya: 900 women & girls (ages 15-59)	Mixed Methods	ND	Cases of vendors capitalizing on socioeconomic vulnerabilities of women and girls by coercing them into sex in exchange for water found to be a common occurrence.
Mukuhlani & Nyamupingidza (2014) [67]	Zimbabwe	Qualitative case study	ND	Water shortages have led to cases of sexual assault at boreholes, with particular risk during times when lines are short (i.e. morning and night).
Varickanickal et al. (2020) [41]	Kenya: 13 women	Photovoice	Jul. 2016	The findings suggest that young women were at risk of sexual abuse when they walk long distances to collect water, especially if they must collect water at night to avoid long lines.

**Table A4:** Demonstrated or estimated effects associated with reducing the time burden associated with water and solid fuel collection and transport.

Study	Housework (including childcare)	Rest and leisure	School Attendance	Income Generation	Health	Relationships	Findings
Aiga et al. (2002) [19]	•	•	ND	•	⦿	ND	Following a water supply intervention, 72.1% (*n* = 145) of participants spent saved time on income generation, leading to a 79% increase in household income.
Ayoade et al. (2015) [28]	ND	ND	⦿	ND	⦿	ND	Out of 800 girls interviewed, 97% reported being late to school, 86% recorded poor school attendance, 21% had experienced injury or neck and back pain from carrying an excessive load of water.
Baker et al. (2018) [20]	ND	ND	ND	ND	⦿	ND	Increased time daily spent collecting household water increased women’s risk of a low-birth weight newborn.
Bisung & Elliot (2017) [56]	•	•	ND	•	ND	ND	An average of 50 min per round trip were saved by a water access intervention. Time-savings were spent on essential household activities and income-generation.
Cooper-Vince et al. (2017) [35]	ND	ND	⦿	ND	⦿	ND	Water insecurity is a risk factor for missed schooling among children in rural Uganda, which is partially mediated by caregiver depression.
Crow et al. (2013) [45]	ND	⦿	⦿	⦿	ND	ND	Water-collection work constrains women’s livelihoods, education and leisure. Less time spent collecting water could allow an increase in preferred activities.
Devoto et al. (2012) [68]	•	•	○	○	○	•	Facilitating access to credit for households to finance private connections can increase time available for leisure and reduce inter and intra-household conflicts on water matters, leading to sustained improvements in well-being.
Garn et al. (2013) [58]	ND	ND	•	ND	ND	ND	Among schools with poor water access during the dry season, those that received a water supply, hygiene promotion and water treatment demonstrated increased enrollment.
Geere et al. (2010) [24]	⦿	⦿	⦿	ND	⦿	ND	Children in rural areas linked both physiological discomfort and time poverty with the burden of water collection in qualitative research settings.
Gibson, et al. (2006) [69]	ND	•	ND	ND	•	ND	Improved water access was associated with three times greater odds of birth in any given month as well as reduced relative risk of child death by 50% per month.
Ho et al. (2014) [70]	ND	ND	ND	ND	⦿	ND	15-min decrease in one-way walk time is associated with 41% reduction in diarrhea prevalence, improved child nutritional status, and 11% reduction in <5 child mortality.
Jagoe at al. (2020) [59]	•	•	ND	•	•	•	Following a cookstove intervention, participants reported spending less time on solid fuel collection and more time on other tasks.
Kelly et al. (2018) [71]	⦿	⦿	⦿	ND	ND	⦿	No significant reductions in school absenteeism from new cook stoves; Qualitatively, there was a reduced burden for collection and preparation of solid fuel.
Nauges & Strand (2013) [18]	ND	ND	⦿	ND	ND	ND	Estimated that a 50% reduction in water carriage time would increase the proportion of girls attending school.
McCray (2004) [72]	ND	ND	ND	ND	⦿	ND	Women, who reported that their ability to collect water was negatively impacted by a need to visit the clinic, were two times more likely to utilize prenatal care services at a low level than other participants.
Mukuhlani & Nyamupingidza (2014) [67]	ND	ND	•	ND	ND	ND	School children spending free time queuing for water instead of attending academic/extracurricular activities.
Pickering & Davis (2012) [3]	⦿	ND	ND	⦿	⦿	ND	The time burden of water collecting has been suggested to influence time spent on income generating activities, child care, and positive health outcomes for children in several settings.
Robson et al. (2013) [25]	ND	ND	⦿	ND	⦿	ND	Efforts to reduce the heavy burden of water carrying on children and especially girls should reduce their ill health and increase their time and energy for school attendance and study.
Rauniyar et al. (2011) [73]	ND	ND	•	○	•	ND	Project reduced drudgery associated with collecting water and improved attendance of high-school-age girls in schools.
Subbaraman et al. (2015) [40]	⦿	ND	⦿	⦿	⦿	ND	58.7% of collectors reported negative impact on work. 28.5% of water collectors reported negative impact on school.
Zolnikov & Blodgett (2016) [38]	•	ND	•	ND	ND	•	Participants’ experiences after water interventions revealed enhanced relationships within household family units; additional personal time was gained and used to rebuild relationships.

• Demonstrated or reported improvements in ability to allot time to desirable alternative activities or health outcomes due to reduced time burden. ⦿ Predicted improvements in ability to allot time to desirable alternative activities or health outcomes due to reduced time burden. ○ No demonstrated or predicted improvements when a particular area was investigated. **ND** No data available for the particular area.
