# Supplementary material for: Diverse Health, Gender and Economic Impacts from Domestic Transport of Water and Solid Fuel: A Systematic Review
Source: Int J Environ Res Public Health. 2021 Oct 1;18(19):10355. doi: 10.3390/ijerph181910355 (PMC8507830; doi:10.3390/ijerph181910355)
Supplement: Supplementary file 1 [file ijerph-18-10355-s001.zip › ijerph-1370239-supplementary.pdf]

International Journal of
Environmental Research and Public Health

MDPI

Supplementary Materials

Table S1. List of Boolean search terms.

Water and solid fuel terms	(*"water transport"* OR *"water procurement"* OR *"water collecting"* OR *"water collection"* OR *"water fetching"* OR *"load carrying"* OR *"water carrying"* OR *"solid fuel collection"* OR *"wood transport"* OR *"water collection time"* OR *"firewood gathering"* OR *"biomass collection"* OR *"fuel procurement"* OR *"fuel preparation"* OR *"firewood bundles"*)
Underserved populations in LMICs	*('africa' OR 'asia' OR 'caribbean' OR 'west indies' OR 'south america' OR 'latin america' OR 'central america' OR 'afghanistan' OR 'albania' OR 'algeria' OR 'angola' OR 'armenia' OR 'armenian' OR 'azerbaijan' OR 'bangladesh' OR 'benin' OR 'byelarus' OR 'byelorussian' OR 'belarus' OR 'belorussian' OR 'belorussia' OR 'belize' OR 'bhutan' OR 'bolivia' OR 'bosnia' OR 'herzegovina' OR 'hercegovina' OR 'botswana' OR 'brasil' OR 'brazil' OR 'bulgaria' OR 'burkina faso' OR 'burkina fasso' OR 'upper volta' OR 'burundi' OR 'urundi' OR 'cambodia' OR 'khmer republic' OR 'kampuchea' OR 'cameroon' OR 'cameroons' OR 'cameron' OR 'camerons' OR 'cape verde' OR 'cabo verde' OR 'central african republic' OR 'chad' OR 'china' OR 'colombia' OR 'comoros' OR 'comoro islands' OR 'comores' OR 'mayotte' OR 'congo' OR 'zaire' OR 'costa rica' OR 'cote divoire' OR 'ivory coast' OR 'cuba' OR 'djibouti' OR 'french somaliland' OR 'dominica' OR 'dominican republic' OR 'east timor' OR 'east timur' OR 'timor leste' OR 'ecuador' OR 'egypt' OR 'united arab republic' OR 'el salvador' OR 'eritrea' OR ethiopia OR fiji OR gabon OR (gabonese AND republic) OR gambia OR gaza OR 'georgia republic' OR (georgia* AND republic) OR 'georgian republic' OR ghana OR 'gold coast' OR grenada OR guatemala OR guinea OR 'guinea-bissau' OR guiana OR guyana OR haiti OR honduras OR india OR maldives OR nauru OR 'papua new guinea' OR indonesia OR iran OR iraq OR 'jamaica' OR 'jordan' OR 'kazakhstan' OR 'kazakh' OR kenya OR kiribati OR 'north korea' OR kosovo OR kyrgyzstan OR kirghizia OR (kyrgyz AND republic) OR kirghiz OR kirgizstan OR 'lao pdr' OR laos OR lebanon OR lesotho OR basutoland OR liberia OR libya OR 'north macedonia' OR macedonia OR madagascar OR 'malagasy republic' OR malaysia OR malaya OR malay OR sabah OR sarawak OR malawi OR nyasaland OR mali OR 'marshall islands' OR mauritania OR mauritius OR 'agalega islands' OR mexico OR micronesia OR moldova OR moldovia OR mongolia OR morocco OR ifni OR mozambique OR myanmar OR burma OR namibia OR nepal OR 'netherlands antilles' OR nicaragua OR niger OR nigeria OR pakistan OR palestine OR paraguay OR peru OR philippines OR philipines OR phillipines OR phillippines OR romania OR rumania OR roumania OR russia OR 'russian federation' OR rwanda OR ruanda OR 'saint lucia' OR 'st lucia' OR 'saint vincent' OR 'st vincent' OR grenadines OR samoa OR 'samoan islands' OR 'navigator island' OR 'navigator islands' OR 'sao tome' OR 'senegal' OR serbia OR montenegro OR 'sierra leone' OR 'sri lanka' OR ceylon OR 'solomon islands' OR somalia OR 'south africa' OR 'south sudan' OR sudan OR suriname OR surinam OR swaziland OR syria OR 'syrian arab republic' OR tajikistan OR tadzhikistan OR tadjikistan OR tadzhik OR tanzania OR thailand OR togo OR 'togolese republic' OR tonga OR tunisia OR turkey OR turkmenistan OR turkmen OR tuvalu OR*

	uganda OR ukraine OR ussr OR 'soviet union' OR 'union of soviet socialist republics' OR uzbekistan OR uzbek OR vanuatu OR venezuela OR vietnam OR 'viet nam' OR 'west bank' OR yemen OR yugoslavia OR zambia OR zimbabwe OR rhodesia OR (developing OR 'less developed' OR 'under developed' OR underdeveloped OR 'middle income' OR 'low income' OR 'underserved' OR 'under served' OR deprived OR poor) AND (country OR countries OR nation OR nations OR population OR world OR economy OR economies)) OR ('low' AND ('gdp' OR gnp OR 'gross domestic' OR 'gross national')) OR ('low and middle income' AND (country OR countries)) OR lmic OR lmics OR 'third world' OR 'transitional country' OR 'global south')
Language and paper type	*[english]/lim AND ('article'/it OR 'article in press'/it OR 'conference paper'/it OR 'conference review'/it OR 'review'/it)*
